# Spatial and temporal distribution of the malaria mosquito *Anopheles arabiensis *in northern Sudan: influence of environmental factors and implications for vector control

**DOI:** 10.1186/1475-2875-8-123

**Published:** 2009-06-07

**Authors:** Tellal B Ageep, Jonathan Cox, M'oawia M Hassan, Bart GJ Knols, Mark Q Benedict, Colin A Malcolm, Ahmed Babiker, Badria B El Sayed

**Affiliations:** 1 Epidemiology Department, Tropical Medicine Research Institute, PO Box 1304, Khartoum, Sudan; 2 Department of Tropical and Infectious Diseases, London School of Hygiene and Tropical Medicine, London, WC1E 7HT, UK; 3 Laboratory of Entomology, Wageningen University and Research Centre, PO Box 8031, 6700 EH, Wageningen, the Netherlands; 4 International Atomic Energy Agency, Seibersdorf Laboratory, A-2444 Seibersdorf, Austria; 5 School of Biological and Chemical Sciences, Queen Mary University of London, London, E1 4NS, UK; 6 National Centre for Research, Ministry of Science and Technology PO Box 2404, Khartoum, Sudan; 7 K&S Consulting, Kalkestraat 20, 6669CP, Dodewaard, the Netherlands

## Abstract

**Background:**

Malaria is an important public health problem in northern Sudan, but little is known about the dynamics of its transmission. Given the characteristic low densities of *Anopheles arabiensis *and the difficult terrain in this area, future vector control strategies are likely to be based on area-wide integrated pest management (AW-IPM) that may include the sterile insect technique (SIT). To support the planning and implementation of future AW-IPM activities, larval surveys were carried out to provide key data on spatial and seasonal dynamics of local vector populations.

**Methods:**

Monthly cross-sectional larval surveys were carried out between March 2005 and May 2007 in two localities (Dongola and Merowe) adjacent to the river Nile. A stratified random sampling strategy based on the use of Remote Sensing (RS), Geographical Information Systems (GIS) and the Global Positioning System (GPS) was used to select survey locations. Breeding sites were mapped using GPS and data on larval density and breeding site characteristics were recorded using handheld computers. Bivariate and multivariate logistic regression models were used to identify breeding site characteristics associated with increased risk of presence of larvae. Seasonal patterns in the proportion of breeding sites positive for larvae were compared visually to contemporaneous data on climate and river height.

**Results:**

Of a total of 3,349 aquatic habitats sampled, 321 (9.6%) contained *An. arabiensis *larvae. The frequency with which larvae were found varied markedly by habitat type. Although most positive sites were associated with temporary standing water around the margins of the main Nile channel, larvae were also found at brickworks and in areas of leaking pipes and canals – often far from the river. Close to the Nile channel, a distinct seasonal pattern in larval populations was evident and appeared to be linked to the rise and fall of the river level. These patterns were not evident in vector populations breeding in artificial water sources away from the river.

**Conclusion:**

The GIS-based survey strategy developed in this study provides key data on the population dynamics of *An. arabiensis *in Northern State. Quantitative estimates of the contributions of various habitat types and their proximity to settlements provide a basis for planning a strategy for reducing malaria risk by elimination of the vector population.

## Background

Malaria is a significant health problem in Sudan, affecting 52% of outpatients and accounting for 9% of all hospitals deaths [1]. Given its diversity and the size of the country, the largest in Africa, it is not surprising that knowledge of malaria transmission dynamics, including the temporal and spatial distribution of vector populations, is generally poor. The Nile valley between Khartoum and the Sudanese/Egyptian border is not one of the most seriously affected areas, but malaria is endemic, and the area is of considerable regional significance and of growing national importance due to large hydroelectric and agricultural development projects.

**Figure 1:**
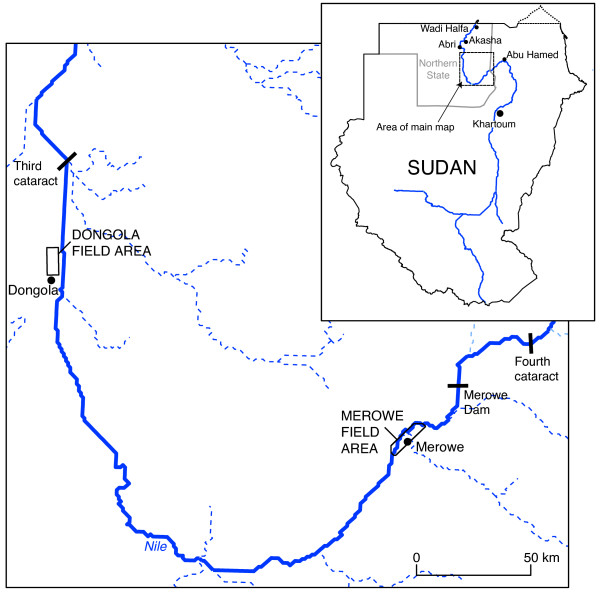
**Location of study sites**. The main channel of the Nile is shown in blue. Seasonal 'wadis' are illustrated by dashed lines.

The area under consideration here is in Northern State, the largest of the 26 states and located in the northwest on the border with Egypt and Libya (Figure 1). Northern State has been prone to significant malaria epidemics at times of very high Nile floods, but one of the most dramatic occurred when the vector, *Anopheles gambiae s.l*., temporarily extended its range into lower Egypt in 1942–3, causing an estimated 139,000 malaria cases and 10,100 deaths [2]. In 2006, malaria accounted for an estimated 17% of hospital admissions in Northern State and was the leading cause of death in hospitals [1].

Results from historical surveys dating back over fifty years, and more recent extensive surveys conducted over the last seven years, have indicated that *Anopheles arabiensis *is common along the Nile valley north of Khartoum to the northern limit of its distribution between Abri and Akasha, about 200 km south of the Egyptian border (Figure 1). In addition to its (probable) incursion into Egypt in the 1940s, *An. arabiensis *has been found on occasion as far north as Wadi Halfa (~250 km north of the current Dongola field area). Historically, it may have had a more northerly distribution, but most, if not all, of the more recent records can be attributed to human assisted dispersal [3,4]. Today, the vector is held in check by poor terrain, less favourable climate and the vector control operations implemented by the joint Egyptian-Sudanese Gambiae Control Project.

Malaria in Northern State presents a difficult control problem that requires an effort disproportionate to the incidence of the disease and the size of the human population at risk. The geographical area affected is not large, but is elongated across difficult terrain with limited accessibility. *Anopheles arabiensis *is the only vector present, and for most of the year, adults are quite difficult to find. The existing control campaign relies heavily on larviciding and source reduction. The use of insecticide to treat adult mosquitoes is often directed to urban areas. This strategy maximizes the number of people protected, but arguably has more impact on biting nuisance caused by *Culex quinquefasciatus *Say than on malaria transmission.

The most focused vector control effort has been directed towards pushing the northern limit of *An. arabiensis *southward along the Nile valley, but forty years of uninterrupted control operations have not moved the boundary much further upstream. The history of vector control along the rest of the Nile within Northern State is patchy, but overall it is clear that the use of the available resources has been largely ineffective. A major contributing factor has been poor understanding of the malaria transmission dynamics and vector bionomics. This is reflected in the limitations of a control programme based largely on insecticide, which is inappropriate for targeting a low-density vector population dispersed widely across difficult terrain.

The vector population is restricted to within a few kilometres of the Nile by the desert, and migration along the river into Northern State is limited by unsuitable terrain along the Abu Hamed Reach. The population is therefore almost entirely isolated, and this has been confirmed by analysis of variation in microsatellite DNA in samples collected along the Nile from Khartoum to the Third Cataract [5]. The vector problem is, therefore, ideally suited to an area-wide integrated pest management (AW-IPM) programme, and particularly to the use of sterile insect technique (SIT) [6].

It is evident from a series of preliminary surveys conducted along the length of the Nile between the Third Cataract and Abu Hammed, that the temporal and spatial distribution of *An. arabiensis *is very dynamic. The Nile flood has always been regarded as of primary significance, but many other factors are evident. To make a decision on appropriate and potentially effective control measures requires a comprehensive understanding of the factors involved and their relative importance. If AW-IPM incorporating SIT is the most suitable and cost effective approach, the detail and accuracy of these data become of paramount importance [7].

In the current study, the distribution of *An. arabiensis *adults was considered too sparse to provide an effective starting point for detailed population studies. A survey of the spatial and temporal distribution of larval breeding sites was more practical, although this was not achievable across the length of the area to be targeted for future AW-IPM. Based on previous surveys, two study zones were selected that as far as possible represented the diversity of terrain and land use found throughout Northern State. This paper reports results from larval surveys conducted at these sites between March 2005 and May 2007. The aim of these surveys was two-fold: to provide a dataset that would aid informed decision making on future malaria control strategies, and to provide a basis for predicting and modelling vector population dynamics in order to facilitate detailed planning for future data gathering and ultimately the design, planning and implementation of the control strategy. Results will also help to address significant knowledge gaps with respect to the epidemiology of malaria in these settings.

## Methods

### Study areas

**Figure 2:**
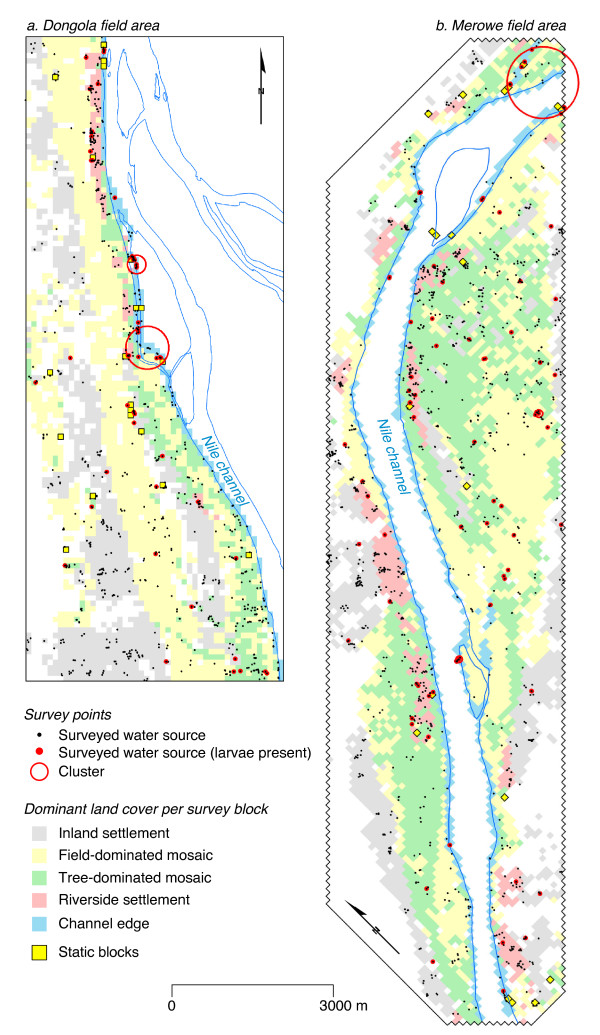
**Maps of the Dongola and Merowe field areas, indicating the locations of surveyed aquatic habitats**. Red dots indicate sites positive for *An. arabiensis *larvae. Red open circles indicate significant clusters of positive sites. RS-derived land cover for each survey block is also shown (white areas indicate bare desert or inaccessible locations and were excluded from the sample universe)

The study was carried out at two field areas adjacent to the River Nile in Northern State, Sudan (Figure 1). The more easterly of the field areas (31.8–31.94 °E, 18.48–18.60 °N; elevation 227–350 m) borders the towns of Merowe and Kareima and is situated about 40 km downstream of the site of the Merowe High Dam. The second field area is located a further 280 km downstream, in the vicinity of Dongola (30.44–30.49 °E, 19.18–19.29 °N; elevation 217–241 m). Surveyed areas were located along the Nile river channel (Figure 2). In Dongola only the western bank of the Nile was included in the surveys because of absence of a bridge and consequent practical problems accessing the eastern bank.

Study areas were selected on the basis of the wide variety of riverbank conditions and land use/land cover types they contained. Ecological conditions within the field sites are therefore considered to be typical of those over a much larger area.

In both field areas, local agriculture is based on irrigation and is primarily influenced by flooding of the Nile and seasonal changes in temperature. River levels are lowest in March and highest in August and September. Date palms dominate much of the cultivated landscape throughout the year, but the area covered by other crops varies seasonally, being greater in the winter when wheat and beans are grown, than in the summer when these are partly replaced by maize and sorghum. Irrigated agriculture, interspersed with settlements, forms a more or less continuous belt along both sides of the Nile channel but rarely extends further than about 4 km away from the river. In non-irrigated areas the dominant land cover is sand, stone desert or scrub vegetation.

### Meteorological and hydrological data

Two solar-powered automatic weather stations (Campbell Scientific; Loughborough, UK) were installed in each field area. Measurements of temperature, relative humidity, wind speed and wind direction were logged at hourly intervals during the period February 2004 to May 2007. Daily data for river level at gauging stations in Kareima and Dongola were obtained from the Ministry of Irrigation, Sudan.

### Land cover mapping

Pan-sharpened multispectral QuickBird data (DigitalGlobe, Longmont, CO, USA) were acquired for January 2004 and January 2005 for the Dongola and Merowe field areas, respectively. Geometric and topographical corrections were carried out using Geomatica OrthoEngine software (v. 9.1; PCI Geomatics, Ontario, Canada) using a combination of 101 ground control points, mapped using differential GPS (Trimble GeoExplorer III, Trimble Navigation Ltd, Sunnyvale, CA, USA), and a high resolution digital elevation model derived by air photogrammetry provided by the Merowe Dam Project [8]. Object-oriented land cover classification was carried out using eCognition software (v. 4; Definiens AG, Munich, Germany). Source images were first 'segmented' into image objects using parameters developed and tested on a small representative sub-region. In addition to standard pixel-level spectral data, object characteristics such as shape, context and texture were then incorporated in the classification process [9,10]. Images were first classified at a high spatial resolution (0.6 m) using 19 predefined land cover types. This classification product was then used to produce a generalized (100 m resolution) map of predominant land cover based on five composite definitions of land cover: 'channel edge', 'inland settlement', 'riverside settlement', 'field-dominated land cover mosaic' and 'tree-dominated land cover mosaic' (Figure 2). Survey blocks not allocated to these classes represented bare desert or waste ground and were not included in the sample universe. For full details of the classification procedures used in this study, including information on adopted class hierarchies, membership functions and classifiers, see [Additional file 1].

### Sampling design

Monthly larval sampling was carried out using 100 m resolution sampling grids generated from the generalized land cover classification products described above. These grids consisted of a total of 2,446 and 4,559 survey cells (or 'blocks') for the Dongola and Merowe field areas respectively. Individual survey blocks covered an area of 1 ha (100 × 100 m). Each month a random sample of 40 blocks, stratified by predominant land cover type, was generated for each field area. Sections of the field area not easily accessible were excluded from the sample, as were survey blocks where the predominant land cover type was bare desert (white areas of Figure 2). In addition to the random blocks, 20 'static' blocks were surveyed in each field area each month. Static blocks were chosen during preparatory field visits and represented locations where a variety of permanent or seasonal water bodies were known to exist.

### Field sampling procedures

Mosquito larva sampling was carried out monthly between March 2005 and May 2007 (full surveys, incorporating random blocks were carried out from May 2005 onwards). For each field visit a map of the static and random survey blocks to be visited was generated and uploaded to Trimble Recon rugged handheld field computers using Trimble PathFinder Office (v. 2.9) and Trimble Terrasync (v. 2.4) mobile GPS/GIS software (Trimble Navigation Ltd). Field computers were connected to Trimble Pocket GPS receivers, enabling fieldworkers to track their vehicle positions in real time and navigate to the target blocks. Within survey blocks, a fieldworker's movements were indicated as a trace on the handheld computer screen, allowing them routinely to assess their coverage of the area to be surveyed [11].

In each survey block all potential aquatic habitats were assessed for mosquito larvae and their locations recorded by GPS. Where possible, 30 dips were carried out at each water source, but in most cases the number of dips was limited by the size of the water body (median number of dips = 10). *Anopheles arabiensis *was identified morphologically and the presence of different larval stages recorded. In subsequent analysis *An. arabiensis *was considered to be present if at least one larva was collected. At each site fieldworkers recorded data on the physical and chemical characteristics of the aquatic habitat (surface temperature, pH, salinity, turbidity, depth, surface area, degree of shading and the presence of aquatic vegetation or algae). Habitat type was assessed on the ground and allocated to one of 20 habitat classes. The general situation of the site in terms of surrounding land use/land cover (*e.g*. cultivated fields, palm groves, settlement, *etc*.) was also recorded. All field data were entered directly into the handheld computer using data entry forms developed in Terrasync Professional software (Trimble Navigation Ltd).

### Data analysis

ArcGIS and ArcInfo Workstation (v. 9.2; ESRI, Redlands, CA, USA) were used to integrate field data with existing GIS and RS data. All statistical analyses were carried out in Stata (v. 10; StataCorp, College Station, TX, USA). A two-sample test of proportions was used to compare frequencies of larva-positive aquatic habitats by field area and by type of survey block (static or random). Bivariate and multivariate logistic regression models were fitted to identify variables associated with the presence of larvae. Multivariate models, incorporating variables significant at p < 0.2 in the bivariate analysis, were developed using a forward-stepwise procedure and assessed using the likelihood ratio test. Interactions between key variables were assessed, and significant terms were included in the multivariate models. All regression analyses were carried out using a combined dataset from random and static blocks.

Kulldorf's spatial scan statistic was used to test whether sites containing *An. arabiensis *larvae were distributed randomly over space, and to identify significant spatial clusters [12]. Analysis was carried out in SaTScan software [13] using a Bernoulli model and a specified upper limit for the window size of 10% of the study population (see [14] for a more detailed explanation of these terms). Spatial analysis was carried out using data from randomly selected survey points (*i.e*. data from static blocks were excluded). Temporal patterns in the proportion of aquatic habitats containing larvae were assessed using basic time series plots. Series were smoothed using a locally weighted polynomial regression (lowess) to filter daily variations and aid visual interpretation of seasonal trends.

## Results

**Figure 3:**
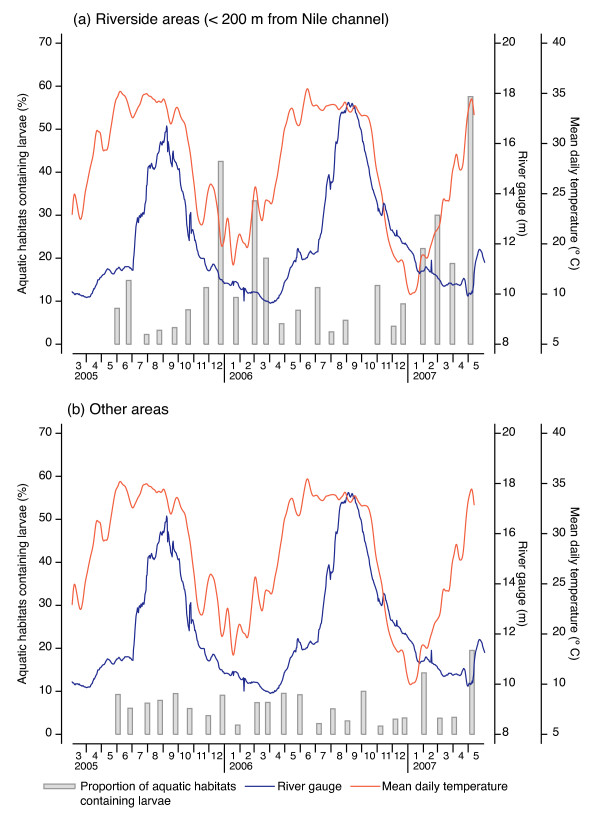
**Temporal patterns in the proportion of aquatic habitats positive for *An. arabiensis *larvae, March 2005 to May 2007**. Results are differentiated for sites within 200 m of the Nile channel (panel a) and other areas (panel b). Corresponding time series for mean daily temperature and river gauge (height) are also indicated

Climatic conditions in Northern State were typically dry and warm to hot, depending on the season. During the period February 2004 to May 2007 the mean monthly temperature for the two field areas combined was 28°C and mean monthly minimum and maximum temperatures were 19.9 and 36.4°C, respectively. The hottest months were May-September, during which maximum daily temperatures consistently exceeded 40°C (Figure 3). The coolest month was January (mean minimum monthly temperature 10.7°C). Average daily diurnal temperature range varied between 14.9°C in August and 18.6°C in March and April. Mean monthly relative humidity was lowest in the period April-June (<18%) and highest in December and January (>36%). No rainfall was observed during the whole study period.

Over a period of 26 months, 52 field surveys were carried out in the Dongola and Merowe field areas. A total of 3,349 aquatic habitats were sampled, of which 321 (9.6%) contained *An. arabiensis *larvae (Table 1). The proportion of sites containing larvae differed significantly between the two field areas (Dongola = 12%, Merowe = 8.1%; p < 0.001). In Dongola the proportion of habitats positive for larvae was higher in static blocks than in random blocks (17.4 *vs*. 8.8%; p < 0.001). No significant difference between the two types of block was observed in Merowe (8.8 *vs*. 7.6%; p = 0.32). Late stage instars (L3+L4) were found in 77% of larvae-positive sites. Pupae were observed in 30% of larvae-positive sites.

**Table 1:** Total number of aquatic habitats sampled and number positive for *An. arabiensis *larvae by type of site and field area

	Dongola		Merowe		Combined	
			
*All sites:*						
Habitats sampled	1,282		2,067		3,349	
Habitats positive for larvae	154	(12.0%)	167	(8.1%)	321	(9.6%)
						
*Random blocks:*						
Habitats sampled	804		1,226		2,030	
Habitats positive for larvae	71	(8.8%)	93	(7.6%)	164	(8.1%)
						
*Static blocks:*						
Habitats sampled	478		841		1,319	
Habitats positive for larvae	83	(17.4%)	74	(8.8%)	157	(11.9%)

### Habitat characteristics

Table 2 lists the types of aquatic habitat encountered in both field areas in descending order of frequency across both field areas. In Dongola the most common water sources encountered were canals (23% of the total sample), followed by containers (including *zirs *– earthenware pots – 10.8%), taps (10.7%) and riverbank (9.3%). In Merowe, containers (26%) and canals (23.5%) were by far the most common types of water sources sampled. The frequency with which larvae were found varied markedly by habitat type. In Dongola, aquatic habitats associated with 'grassy knolls' (soil terraces close to the main river channel), *khors *(seasonal tributary channels), leaking underground pipes, riverbanks, brickworks, seepage from canals and leaking surface pipes were most often positive for larvae. All wells and residue flood water pools sampled were positive for larvae, but together constituted a very small sample (Table 2). Larvae were absent from containers, cisterns and leaking water tanks and were found only rarely in canals, flooded fields, domestic drains, taps or culverts. A broadly similar picture emerges in Merowe, where habitats associated with grassy knolls, riverbanks, leaking underground pipes, brickworks, *khors *and leaking canals were most likely to contain larvae. Unlike Dongola, all wells in the Merowe field area were unproductive. Note that Table 2 represents a combined dataset including both random and static blocks. Separate analyses for static and random block showed similar patterns.

**Table 2:** Types of aquatic habit sampled and number/proportion positive for larvae

	Dongola			Merowe			Combined		
	
Habitat type*	No. sampled	No. positive	% positive	No. sampled	No. positive	% positive	No. sampled	No. positive	% positive
Container	138	0	0.0	536	5	0.9	674	5	0.7
Canal (flowing)	167	2	1.2	273	2	0.7	440	4	0.9
Canal (stagnant)	130	3	2.3	213	11	5.2	343	14	4.1
Flooded field	67	2	3.0	164	4	2.4	231	6	2.6
Domestic drain	69	2	2.9	146	8	5.5	215	10	4.7
Tap	137	2	1.5	65	5	7.7	202	7	3.5
Riverbank	119	35	29.4	82	42	51.2	201	77	38.3
Water tank	1	0	0.0	144	8	5.6	145	8	5.5
Underground pipe	64	23	35.9	77	16	20.8	141	39	27.7
Cistern	58	0	0.0	53	1	1.9	111	1	0.9
Brickworks	54	15	27.8	53	14	26.4	107	29	27.1
Canal seepage	79	19	24.1	27	6	22.2	106	25	23.6
Animal water pool	31	2	6.5	58	12	20.7	89	14	15.7
Surface pipe	24	5	20.8	58	7	12.1	82	12	14.6
Khor	7	3	42.9	67	15	22.4	74	18	24.3
Culvert	47	2	4.3	8	3	37.5	55	5	9.1
Grassy knoll	50	30	60.0	5	2	40.0	55	32	58.2
Underground pump	35	5	14.3	16	3	18.8	51	8	15.7
Flood pool	1	1	100.0	11	1	9.1	12	2	16.7
Well	3	3	100.0	8	0	0.0	11	3	27.3

All habitats	1,281	154	12.0	2,064	165	8.0	3,345	319	9.5

*Excludes four sites where habitat type was not recorded

During field surveys, sites were also classified according to surrounding situation or land cover (see Table 3). The largest number of aquatic habitats was found in areas of settlement (1325, or 40% of all habitats visited), but fewer than 5% of these sites contained larvae. Similarly, of more than one thousand aquatic habitats surveyed within palm groves, only 6% contained larvae. Positive sites were most likely to be found in areas constituting channel edge (n = 370; 35% of sites positive) and within brickworks (n = 89; 19% of sites positive).

**Table 3:** Observed situation (land cover) of sampled aquatic habitats

	Dongola			Merowe			Combined		
Situation type*	No. sampled	No. positive	% positive	No. sampled	No. positive	% positive	No. sampled	No. positive	% positive

Settlement	429	27	6.3	896	34	3.8	1,325	61	4.6
Palm grove	374	17	4.5	718	50	7.0	1,092	67	6.1
Channel edge	188	69	36.7	182	60	33.0	370	129	34.9
Bare fields	123	17	13.8	184	15	8.2	307	32	10.4
Cultivated fields	117	12	10.3	41	2	4.9	158	14	8.9
Brickworks	51	12	23.5	38	5	13.2	89	17	19.1

All situations	1,282	154	12.0	2,059	166	8.1	3,341	320	9.6

*Excludes eight sites where situation was not recorded

### Logistic regression modelling for larvae presence/absence

#### Water body characteristics

Bivariate logistic regression models combining presence of *An. arabiensis *and various water body characteristics are presented in Table 4. In both field areas the presence of larvae was more likely in the following situations: in water bodies larger than 1 m^2^; in moderately saline water; in water of pH > 6.5; and in relatively un-shaded sites. The presences of algae or vegetation were significant risk factors for the presence of larvae in both field areas. In Merowe, larvae were more likely to be found in non-turbid water, but this distinction was not observed in Dongola. In Merowe, larvae were less common in deep water (depth > 50 cm), but again this effect was not evident in Dongola. Water temperature was not a significant determinant of risk of larval presence in either field area. For both field areas, minimum adequate models developed using multivariate logistic regression retained all significant variables (p < 0.001) in Table 4. In other words, none of the covariates significant in the bivariate analysis became non-significant when controlling for other covariates.

**Table 4:** Bivariate logistic regression results for presence of *An. arabiensis *larvae: water body characteristics

	Dongola				Merowe			
		
	Habitats sampled	OR*	95% CI	P value†	Habitats sampled	OR	95% CI	P value†
*Water temperature (°C)*				**0.26**				**0.87**
<24	366	1.00			533	1.00		
24–27.9	281	1.01	0.63–1.63	0.95	400	1.10	0.68–1.79	0.70
28–31.9	376	0.76	0.47–1.20	0.24	569	1.11	0.71–1.74	0.63
32+	258	1.23	0.77–1.96	0.38	565	1.23	0.79–1.90	0.35

*Salinity (%)*				**<0.0001**				**<0.0001**
0	318	1.00			890	1.00		
0.01–0.25	689	5.02	2.66–9.48	<0.0001	807	2.88	1.95–4.26	<0.0001
>0.25	274	4.49	2.25–8.98	<0.0001	370	2.49	1.56–3.99	<0.0001

*pH*				**<0.0001**				**0.0005**
< 6.5	253	1.00			424	1.00		
6.5	756	1.00	0.62–1.63	0.99	1,358	1.05	0.68–1.61	0.83
> 6.5	272	2.59	1.55–4.31	<0.0001	285	2.29	1.39–3.78	0.001

*Water turbidity*				**0.54**				**0.0002**
Non-turbid	871	1.00			1,141	1.00		
Turbid	410	0.89	0.62–1.29		926	0.53	0.38–0.75	

*Water depth (cm)*				**0.20**				**<0.0001**
<10	844	1.00			1,148	1.00		
10–50	325	0.94	0.64–1.39	0.76	418	1.39	0.97–1.99	0.07
>50	112	0.53	0.25–1.12	0.10	501	0.32	0.18–0.55	<0.0001

*Surface area (m^*2*^)*				**0.0001**				**<0.0001**
<1	552	1.00			947	1.00		
1–10	438	1.97	1.36–2.86	<0.0001	619	3.86	2.62–5.68	<0.0001
>10	291	0.79	0.48–1.32	0.37	501	1.81	1.14–2.87	0.01

*Degree of shading (%)*				**0.0008**				**<0.0001**
<25	888	1.00			1,290	1.00		
25–75	285	0.56	0.35–0.89	0.01	546	0.67	0.45–0.97	0.04
>75	108	0.29	0.12–0.73	0.01	231	0.12	0.04–0.38	<0.0001

*Presence of algae*				**<0.0001**				**<0.0001**
No	1,027	1.00			1,701	1.00		
Yes	254	3.62	2.54–5.17		366	5.81	4.18–8.07	

*Presence of vegetation*				**<0.0001**				**<0.0001**
No	598	1.00			1,042	1.00		
Yes	683	2.18	1.52–3.13		1,025	2.27	1.62–3.17	

* Odds ratio

† P values in bold are from likelihood ratio tests (overall significance of exposure variables); for variables with multiple exposure levels P values from Wald tests are also reported.

#### Landscape characteristics

Two measures of land cover in the vicinity of aquatic habitats were generated in this study (Table 5). The first is a field-based assessment of situation or surrounding land cover type; the second corresponds to the RS-derived aggregated land cover class allocated to each 100 × 100 m survey block. The two classification systems, although not identical, are broadly comparable. For directly-observed land cover, and using 'bare fields' as a reference class, the presence of land cover types associated with the edge of the Nile channel (including rock pools, sand and mud banks, *khors *and grassy knolls) constituted the single strongest risk factor for the presence of larvae (for the two field areas combined, OR = 4.6; 95% CI = 3.0–7.0; p < 0.0001). Presence of settlement was associated with relatively low risk of larvae in both field areas. Risk of larvae in cultivated areas or brickworks did not differ significantly from the reference class in either field area. In Dongola, palm groves were associated with a lower risk of larvae than the reference class, but no similar significant effect was observed in Merowe.

**Table 5:** Bivariate logistic regression results for presence of *An. arabiensis *larvae: landscape characteristics

	Dongola				Merowe			
	
	Habitats sampled	OR	95% CI	P value*	Habitats sampled	OR	95% CI	P value*
*Observed land cover*				**<0.0001**				**<0.0001**
Bare fields	123	1.00			184	1.00		
Brickworks	51	1.92	0.84–4.38	0.122	38	1.71	0.58–5.02	0.33
Cultivated fields	117	0.71	0.32–1.56	0.399	41	0.58	0.13–2.63	0.48
Palm grove	373	0.30	0.15–0.60	0.001	718	0.84	0.46–1.54	0.58
Channel edge	188	3.62	2.00–6.53	<0.0001	182	5.54	3.01–10.22	<0.0001
Settlement	429	0.42	0.22–0.80	0.008	896	0.44	0.24–0.83	0.01

*RS-derived land cover*				**<0.0001**				**<0.0001**
Inland settlement	361	1.00			316	1.00		
Tree-dominated mosaic	225	0.79	0.35–1.80	0.58	305	3.28	1.67–6.46	0.001
Field-dominated mosaic	220	1.00	0.46–2.17	0.99	598	1.67	0.86–3.25	0.13
Riverside settlement	248	4.46	2.52–7.88	<0.0001	609	0.99	0.49–2.03	0.99
Channel edge	227	8.32	4.79–14.45	<0.0001	234	8.35	4.36–15.97	<0.0001

*Distance to river (m)*				**<0.0001**				**<0.0001**
< 200	370	1.00			564	1.00		
200–399	178	0.66	0.41–1.05	0.08	445	0.54	0.34–0.84	0.01
400–799	127	0.40	0.21–0.75	0.004	543	0.34	0.21–0.55	<0.0001
800+	606	0.19	0.12–0.29	<0.0001	515	0.83	0.56–1.22	0.34

* P values in bold are from likelihood ratio tests; P values for individual exposure categories are from Wald tests.

Within the RS-derived land cover classification areas of settlement were arbitrarily sub-divided into localities within or outside a 200 m buffer of the Nile channel ('riverside settlement' and 'inland settlement' respectively). Using inland settlement as a reference class, areas close to the channel edge were associated with a greatly elevated risk of larvae being present (for the two field areas combined, OR = 8.2; 95% CI = 5.4–12.5; p < 0.0001). In Dongola, areas of riverside settlement were significantly more likely to contain larvae than areas of settlement 'inland' – but no significant difference in risk between the two classes was apparent in Merowe. In Merowe, land primarily given over to cultivation ('field-dominated mosaic') was more likely to contain larvae than the reference class, although the significance of this difference was borderline, and no similar effect was observed in Dongola.

To evaluate the overall effect of distance from river on presence of larvae (*i.e*. regardless of local land cover), an additional logistic regression model was developed on the basis of distances between each aquatic habitat and the Nile channel, calculated in a GIS (Table 5). Using habitats within 200 m of the channel as a reference class, in Dongola there is a clear and monotonic decline in risk with increasing distance from the Nile (and particularly for distances > 400 m). In Merowe the relationship between risk and distance to channel appears to be more complex -there being no significant increase in risk in areas greater than 800 m from the river.

### Spatial distributions of larvae

The spatial distributions of surveyed sites in the two field areas are shown in Figure 2, which also shows the land use categories used to stratify the random survey blocks in each site. To assess whether distinct spatial clusters in the distribution of breeding sites exist, a spatial scan statistic was determined for randomly-selected sites separately for Dongola and Merowe. In Dongola, two clusters consisting of 16 breeding sites (expected = 2.3; relative risk = 8.7; p < 0.001) and 18 breeding sites (expected = 3; relative risk = 7.7; p < 0.001) were identified (Figure 2a). In Merowe (Figure 2b), one major cluster containing 12 breeding sites (expected = 1.9; relative risk = 7.1; p < 0.001) was identified at the north of the field area. Four very small clusters, each containing between four and seven breeding sites were also identified.

### Seasonal distributions of larvae

Bar charts in Figure 3 show seasonal patterns in the proportion of aquatic habitats positive for *An. arabiensis *larvae, together with temporal variations in mean daily temperature and mean daily river gauge (river height) over the two field areas. Distinct seasonal patterns are evident, particularly in areas situated within 200 m of the main channel (Figure 3a) where decreased river height appears to promote breeding. Seasonal variations in areas away from the river are less marked, and percentages of larvae-positive sites are generally much lower in these areas, compared with sites within 200 m of the main channel. (Note however, that the total number of positive habitats found in inland and at riverside sites was roughly similar due to the much larger number of sites sampled inland). During very high river levels (August to November), the proportion of larvae-positive sites was higher in areas away from the river.

Seasonal patterns in the proportion of habitats containing larvae differed between years. In 2005, for example, the level of the Nile fell rapidly from mid-September and by December the river gauge, averaged across the two sites, was 10.8 m and the proportion of aquatic habitats in riverside areas containing *An. arabiensis *larvae was above 40% (Figure 3a). In 2006, river levels appear to have dropped off relatively slowly in the period October-December; the average river gauge in December was 1.3 m higher than it had been twelve months previously and the proportion of habitats positive for larvae in areas near the Nile was only about 10%.

## Discussion

Overall, analysis of survey results by habitat type and situation suggests that a large proportion of breeding sites were associated with riverside habitats. Across both field areas, 40% of all larvae sampled were from breeding sites located within 100 m of the Nile channel. This finding is consistent with those of other studies of vector breeding in the vicinity of major streams or rivers [15-17]. However, a significant number of positive sites were located away from the river (see Figure 2) and were associated largely with artificial habitats such as leaking pipes and brickworks.

Temporal patterns in the proportion of aquatic habitats containing larvae (Figure 3) are consistent with those previously reported by Dukeen and Omer [3] for the Nile valley north of Dongola. Dukeen and Omer were able to sample larvae in riverside locations throughout the period November to June, but found that from July increasing river levels quickly reduced the number of suitable habitats until, by August, very few pre-existing breeding sites had escaped inundation. Observed densities of *An. arabiensis *larvae and adults declined rapidly in riverside areas from July onwards, although immature stages continued to be found in water sources away from the Nile channel. This picture is entirely consistent with the results from the current study: in riverside areas the proportion of aquatic habitats containing larvae was lowest in July to October, but no similar pattern was observed in aquatic habitats located away from the river and therefore not influenced by river height.

In riverside areas larval populations appear to increase rapidly once river levels begin to fall significantly in November. Pools in riverbank areas, left behind by the receding channel, are most common at this time of year and it seems likely that it is this increased availability of suitable larval habitats for *An. arabiensis *that is most important in determining larval density and adult abundance. The fact that these months coincide with a period of relatively cool daytime temperatures and relatively humid conditions is also likely to be highly significant in terms of adult survivorship and vectorial capacity [2]. Data for malaria admissions in Northern State suggest case numbers peak in May, and this is consistent with the population dynamics of the vector described here.

A detailed appraisal of the results reported here will help to define the future of vector control in Northern State, but a discussion of two non-exclusive options may suffice to illustrate the critical importance of the current study. The main findings confirm the importance of naturally occurring riverside larval breeding sites and their seasonal predictability, but non-persistence. In contrast, artificial breeding sites appear to have a less predictable spatial pattern, and while the overall contribution to the total population is much lower, the sites can be present at any time of the year and may be long-lasting. This suggests that a two-pronged strategy may be most appropriate: a short seasonal but intense control effort launched from the river combined with a year-long, less intense offensive in and around the villages approached from the roads and desert tracks.

A massively more effective and efficient control programme than the current one based on larviciding and source reduction would be one option. The logistics of such a programme would be challenging and very costly. The use of boats to access riverside breeding sites is in fact the preferred strategy adopted by the Gambiae Control Project north of the Third Cataract, but it is difficult to conceive how this could be achieved at the necessary scale. The riverside breeding sites appear and disappear in quick succession; treated sites may be replaced by new nearby sites in a matter of days. Workers would need to visit all areas along a roughly 500 km reach of the Nile every week for up to two months and be prepared to search for sites within at least 200 m of the riverbank. This could be dealt with a by a smaller task force and through education of residents. Access is, however, only part of the problem. There is also still the inefficiency of transporting and applying insecticide to target a low-density population and the issues of environmental hazards and impact on non-target species.

SIT may offer an attractive alternative that overcomes most of the disadvantages of insecticides. The discovery that most larval breeding sites occur within 500 m of the river suggests that delivery of sterile males from boats would effectively cover most of the target area without the need for workers to move far from the riverbank. On the other hand, it is likely that the non-seasonal larval breeding sites located further from the river would seriously undermine this strategy. Strategic planning should therefore extend to a review of alternative delivery methods and their integration within the programme.

This study illustrates the potential benefits of using geospatial tools (GIS, RS, GPS) for planning and carrying out entomological surveys. Through their use it was possible to rapidly assemble a detailed and extensive dataset on *An. arabiensis *breeding sites using relatively limited resources. In terms of data representativeness, the stratified random sampling incorporated in this study has clear advantages over more orthodox 'purposeful' sampling commonly adopted in other studies.

The combination of the current dataset and remotely sensed data for the whole field site will help provide a comprehensive picture of the vector population, but some limitations to this approach were revealed. For example the association of larval breeding sites with poorly maintained pipes, pumps and irrigation channels is not something that can be easily extended outside of the study areas. Pipes can be associated with houses, but not all houses have plumbing, not all houses are occupied and many areas practice water conservation throughout much of the year by limiting availability to certain hours of the day. Therefore, future studies will need to identify alternative ways of capturing this information.

How critical the non-seasonal larval breeding sites are to sustaining the Northern State *An. arabiensis *population also has to be addressed. The low numbers of vectors away from the river may act as the seedbed for large increases in the population when the level of the Nile falls. This is not a new observation – in 1945 Shousha reported that: "during most of the year there are relatively few breeding places for *An. gambiae *in areas of basin irrigation. However, there are enough to produce a quantity of *An. gambiae *sufficient to cause very serious epidemics of malaria" [2]. If that is true, it suggests that a focused effort on the elimination of larval breeding sites during the middle of the year would have a dramatic impact on the overall population.

## Conclusion

• Integrative geospatial analysis using GIS, RS and GPS enabled rapid and effective assembly of entomological data to aid the design of an AW-IPM vector elimination programme incorporating SIT.

• The majority of *An. arabiensis *breeding is associated with the main Nile River channel and subject to its seasonal fluctuations. Larval numbers fall with increasing river level due to flushing of breeding sites; the opposite occurs with falling levels, when numerous breeding sites are created and provide optimal habitat.

• AW-IPM necessitates coverage of inland (man-made) sites in which breeding occurs year-round; such sites constitute the focal points from which populations build up when the Nile provides suitable habitat.

• An SIT campaign will benefit from targeted elimination of inland breeding sites during the hot dry season (July-October), followed by sterile male releases during the more conducive season (November onwards).

## Competing interests

The authors declare that they have no competing interests.

## Authors' contributions

All authors contributed in developing the idea of the study, participated in the selection of the study sites and contributed to the final paper. JC designed the larval sampling strategy, carried out associated image processing and led the data analysis. TBA was responsible for carrying out field surveys and managing the vector dataset.

## Supplementary Material

Additional file 1 **'Additional documentation: digital image processing of QuickBird data for land cover mapping'**. Document provides details of steps involved in producing classifications of land use/land cover using digital satellite data. Click here for file
