# Supplementary material for: Spatial and temporal distribution of the malaria mosquito Anopheles arabiensis in northern Sudan: influence of environmental factors and implications for vector control
Source: Malar J. 2009 Jun 7;8:123. doi: 10.1186/1475-2875-8-123 (PMC2698915; doi:10.1186/1475-2875-8-123)

Additional documentation: digital image processing of QuickBird data for land cover mapping

Because of the combination of high spatial resolution and low spectral resolution associated with QuickBird imagery it is often difficult to apply traditional classification algorithms (which use spectral properties of individual image pixels) to derive thematic information, particularly in heterogeneous environments. Object-based approaches to classification, which attempt to describe relationships in terms of several categories of object characteristics (in addition to pixel-level information), have generally been shown to yield better classification results in these cases. This document describes the object-oriented approach to land cover classification used in the current study. All analyses were carried out using eCognition v. 4 (Definiens AG, München, Germany).

Image segmentation

In order to classify objects, source images must be partitioned using a segmentation routine. This process extracts meaningful image objects (*e.g.* streets, houses, vegetation patches) based on their spectral and textural characteristics. In eCognition, segmentation is a semi-automated process where the user can define specific parameters that influence size and shape of the resulting image segments. The resulting objects are attributed not only with spectral statistics, but also with shape and contextual information, relationships with neighbouring objects and texture parameters. In the current analysis a hierarchical approach to segmentation was employed, in which three 'levels' were defined using the parameters listed in Table 1. These parameters were developed subjectively from a test region representing a 4 × 1 km transect orthogonal to the Nile channel. Results of the image segmentation are shown for a small portion of the test area in Figure 1.

Table 1. Parameters used for segmentation levels 1-3

Segmentation level	Scale parameters	Homogeneity criteria		Shape ratio	
		Colour	Shape	Compactness	Smoothness
1	10	0.7	0.3	0.4	0.6
2	25	0.7	0.3	0.4	0.6
3	1000	0.7	0.3	0.4	0.6

Segmentation at levels 1 and 2 was carried out by equally weighting of all four QuickBird bands and using relatively small scale parameters. The third segmentation level, which represents the pre-defined 100 × 100 m sampling grid for the two field areas, was carried out on the basis of a GIS thematic layer only, with all image layer weights set to zero.

Because of the high spatial resolution of the QuickBird data (and the resulting large files sizes), carrying out segmentation on the either the whole of each image mosaic, or even large portions of each mosaic, in a single operation was not possible computationally. Instead, the Dongola and Merowe mosaics were split into a number of smaller image segments (comprising 19 and 50 separate sub-images in each case), and

segmentation and subsequent classification were carried out on each segment individually.

Image classification

Following the segmentation process, image classification and analysis in eCognition makes use of segmented image objects, rather than individual pixels. Classification is based on user-defined fuzzy class descriptions of spectral and spatial features. eCognition generally uses a nearest neighbour algorithm (class assignment based on minimum distance measures), but the classification process can also include a variety of different types of information, including measures of object texture, context and shape.

The class hierarchy used for land cover classification in this exercise is shown in Figure 2. The hierarchy has two levels (named level 2 and level 3 in this case to correspond with the relevant segmentation levels as set out in Table 1). Level 2 incorporates 19 individual land cover classes, many of which are organised into larger ‘group’ classes (*e.g.* vegetated fields, which consists of five child classes). Level 2 classes were predominantly defined on the basis of spectral information (nearest neighbour distance)[1], using training sets developed for each of the 69 image subsets individually. In addition to using the nearest neighbour classifier, class-specific membership functions were developed for the following classes:

Alluvium (all classes): function based on distance to neighbouring water objects

Bare fields (wet and dry): function based on distance to neighbouring water objects

Vegetated fields (all classes): function based on distance to neighbouring shadow objects

Water shadow: function based on relative border with mud bank, sandbank and water objects

Trees (all classes): relative area of bright bare ground objects within 20 m; relative area of ‘other’ shadow objects within 10 m

These membership functions were included because, when using only spectral indices, a significant amount of inter-class confusion occurred between (a) bare fields and alluvium; (b) shadows generated by buildings or trees and those generated by river bank; and (c) certain tree and vegetated field types.

Classification of Level 2 objects yielded a fairly detailed land cover classification for each of the image segments in the two field areas (see example in Figure 3). This classification output was then used as an input for Level 3 classification.

---

[1] In this case the standard definition for nearest neighbour included object spectral means for all bands, ratios (the mean value for an object divided by the sum of all spectral mean values) for bands 1 and 4, mean absolute difference to neighbour objects for band 1 and maximum difference.

Main parent Classes at Level 3 were ‘riverside’ (sampling grid cells containing >10% alluvium water or water shadow objects) and ‘non-riverside’. The ‘non-riverside’ class was then divided into child classes representing ‘riverside settlement’, ‘inland settlement’ and ‘non-settlement’ using a set of membership functions relating to average spectral difference between neighbours of sub-objects, minimum values in band 4, relative area of tree objects, relative area of ‘other’ shadow objects and total area if ‘bare’ objects. Settled areas were divided into riverside and inland classes using an arbitrary distance cut-off of 400 m from riverside objects. The ‘non-settlement’ class was further sub-divided into three remaining classes representing land cover mosaics dominated by fields, trees, or ‘other’.

As illustrated by the example in Figure 5, the output of the Level 3 classification was a set of 59 images consisting of 100 × 100 m pixels (corresponding to the pre-defined sampling grid), within which each pixel had a unique value corresponding to one of the following cover classes:

Riverside
Inland settlement
Riverside settlement
Field-dominated land cover mosaic
Tree-dominated land cover mosaic
Other

The final step in the classification process was to reassemble the individual sub-images into mosaics for each field area.

Figure 1. Results of object segmentation at Levels 1-3 for a 400 × 300 m portion of the Dongola field area

Segmentation level 1

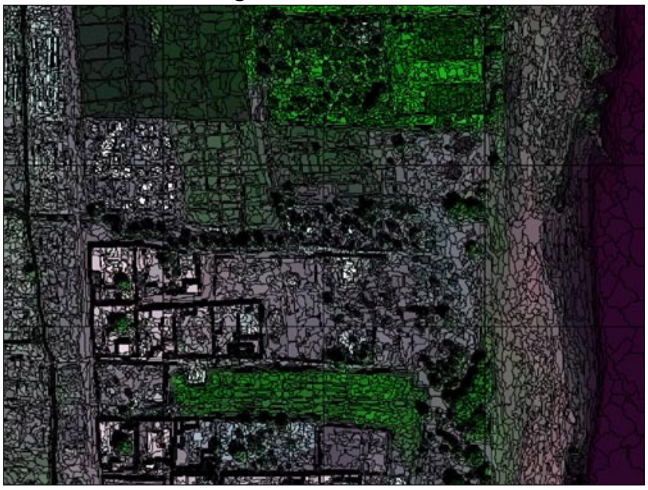

Segmentation level 2

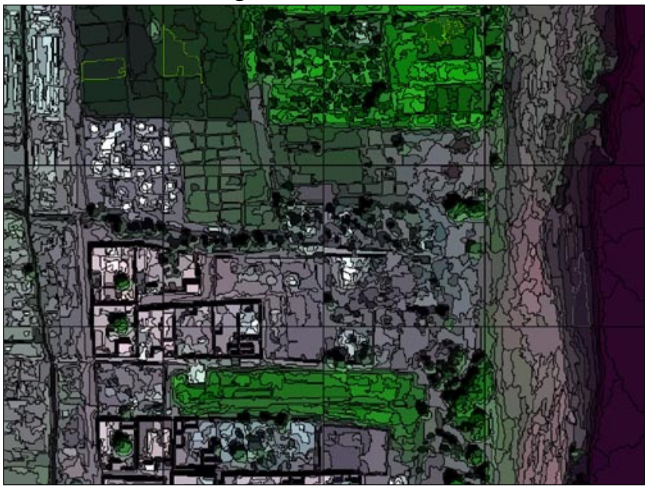

Segmentation level 3

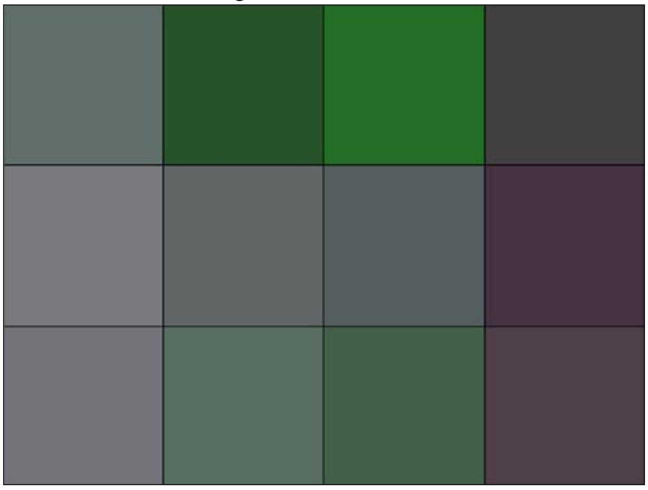

Figure 2. Class hierarchy used for land cover classification

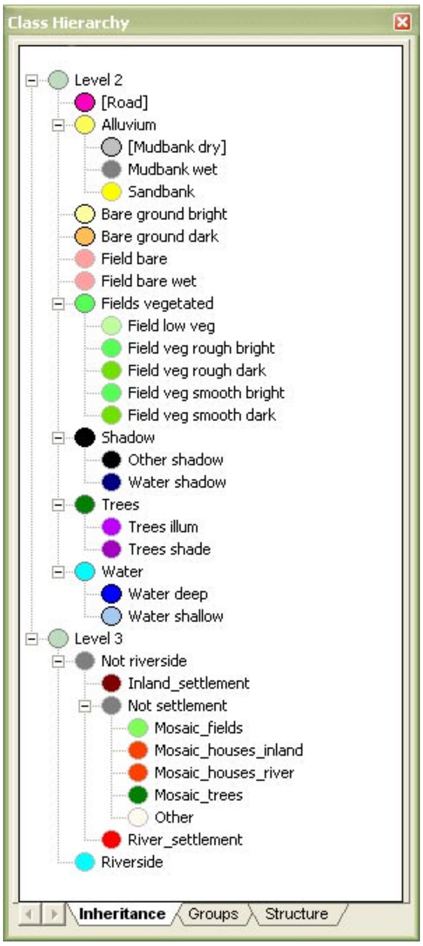

Figure 3. Classified output for segmentation object levels 2 and 3 for one image subset (image size 1.1 × 1.6 km) in the Dongola field area

Level 2

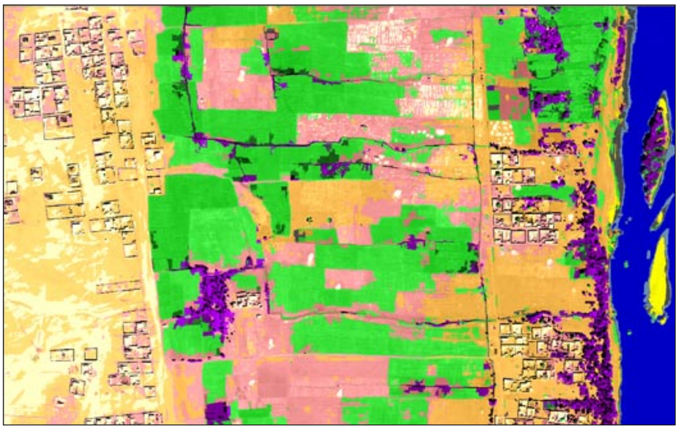

Level 3

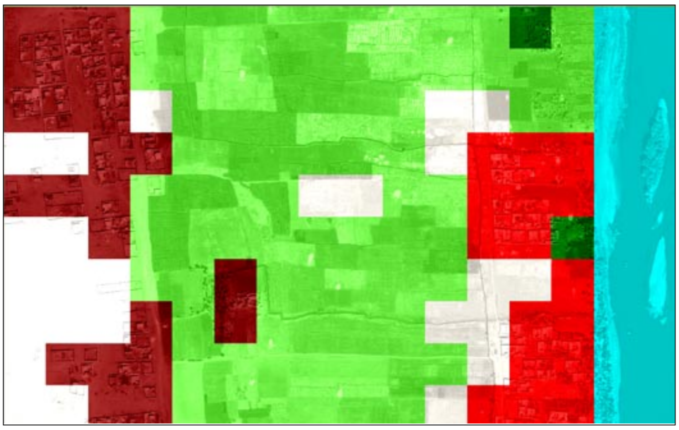

Supplement: Additional file 1 — 'Additional documentation: digital image processing of QuickBird data for land cover mapping'. Document provides details of steps involved in producing classifications of land use/land cover using digital satellite data. [file 1475-2875-8-123-S1.pdf]
